# Fournier gangrene is associated with increased length of stay and higher healthcare costs compared to non-perineal necrotizing soft tissue infections: a retrospective analysis of the National Inpatient Sample (2016–2020)

**DOI:** 10.1017/ash.2025.10084

**Published:** 2025-08-07

**Authors:** Hayato Mitaka, Kristen McQuerry, Kelsey Karnik, Alexandre R. Marra, Toshio Naito, Patrick Ten Eyck, Paul G. Auwaerter, Yuji Yamada, Takaaki Kobayashi

**Affiliations:** 1 Department of Medicine, University of Colorado School of Medicine, Aurora, CO, USA; 2 Department of Biostatistics, College of Public Health, University of Kentucky, Lexington, KY, USA; 3 Faculdade Israelita de Ciências da Saúde Albert Einstein, Hospital Israelita Albert Einstein, São Paulo, SP, Brazil; 4 Department of Internal Medicine, University of Iowa Health Care, Iowa City, IA, USA; 5 General Medicine, Juntendo University Hospital, Tokyo, Japan; 6 Institute for Clinical and Translational Science, University of Iowa, lowa City, IA, USA; 7 The Sherrilyn and Ken Fisher Center for Environmental Infectious Diseases, Johns Hopkins University School of Medicine, Baltimore, MD, USA; 8 Brookdale Department of Geriatrics and Palliative Medicine, Icahn School of Medicine at Mount Sinai, New York, NY, USA.; 9 Division of Infectious Diseases, Department of Internal MedicineUniversity of Kentucky College of Medicine, Lexington, KY, USA

## Abstract

**Background:**

Fournier gangrene (FG) is a necrotizing soft tissue infection (NSTI) of the perineum. Recent retrospective studies from quaternary centers suggest improved outcomes and a potentially less aggressive clinical course for FG than non-perineal NSTIs. However, comprehensive nationwide data remain limited.

**Methods:**

This retrospective cohort study analyzed the National Inpatient Sample (2016–2020) to compare outcomes between FG and non-perineal NSTIs. Adult patients undergoing surgical debridement with a diagnosis of FG or NSTI were identified using ICD-10 codes. Outcomes included in-hospital mortality, length of stay (LOS), hospital costs, and home discharge rates. Multivariable regression analyses adjusted for patient demographics, comorbidities, and hospital characteristics.

**Results:**

A total of 5,007 FG and 24,782 non-perineal NSTI patients were identified. Crude in-hospital mortality rates were 5.8% for FG and 5.4% for non-perineal NSTIs, with stable trends observed over five years. After adjustment, no significant difference in mortality was observed (adjusted odds ratio [aOR]: 1.04; 95% CI: 0.90–1.20). However, FG was associated with longer LOS (adjusted mean difference: 1.99 days; 95% CI: 1.53–2.46) and higher hospital costs ($37,809 higher; 95% CI: $29,540–$46,077). Home discharge rates were similar between groups (aOR: 0.97; 95% CI: 0.89–1.05).

**Discussion:**

Despite similar mortality rates, FG hospitalizations were associated with increased LOS and higher costs compared to non-perineal NSTIs. These findings may suggest potential nationwide disparities in FG care quality, particularly outside specialized referral centers. Further research is needed to understand if standardized care pathways tailored to FG may optimize management and reduce resource utilization.

## Introduction

Fournier gangrene (FG) is a necrotizing soft tissue infection (NSTI) of the perineum historically associated with significant mortality rates of 20%–40%.^1–3^ The treatment of NSTIs, including FG, is complex and requires early surgical debridement of necrotic tissues, prompt initiation of broad-spectrum antibiotics, and critical care focused on hemodynamic stabilization and management of organ failure.^4^ Multiple surgical procedures may be necessary for FG during the hospital stay, including repeated debridement, complex wound closures, and urinary or fecal diversions.^5^ Patient transfers to a tertiary referral center are frequent to ensure optimal care from a multidisciplinary team.^5,6^ Despite these treatments, FG has been associated with significant morbidity and mortality, with about a quarter of patients requiring skilled nursing care on discharge.^5^

However, recent evidence suggests that the clinical outcomes of FG may have improved substantially over the past decades and could be more favorable than those of NSTIs involving other anatomical sites. For instance, a single-center study at a quaternary referral center reported a mortality rate of 3.6% for FG between 2012 and 2015,^7^ in contrast to the higher mortality rates of 9%–22% observed for NSTIs overall at three other academic medical centers from 2009 to 2014.^6^ Similarly, registry data from another quaternary referral center indicated lower odds of mortality and shorter antibiotic durations (8.3 d vs 10.6 d) for perineal NSTIs compared to non-perineal NSTIs.^8,9^ Furthermore, old population-based studies utilizing the National Inpatient Sample (NIS) database provide additional evidence supporting the notion of a more favorable clinical trajectory for FG. Two independent analyses of NSTIs reported median lengths of stay (LOS) of 9 days for FG versus 11–12 days for NSTIs overall.^5,10^ Collectively, these findings may suggest that FG may represent a distinct clinical entity characterized by lower mortality and a less aggressive course compared to NSTIs at other sites.^8,9^

These observations raise the possibility that institutional guidelines or clinical pathways explicitly tailored for FG, including considerations for shorter antibiotic courses, may be appropriate if FG consistently demonstrates a less severe clinical course. However, current evidence remains largely hypothetical, as prior database studies did not directly compare FG outcomes to those of non-perineal NSTIs. Additionally, the generalizability of favorable outcomes from single-center observational studies is limited, making it difficult to draw broad conclusions. In this study, we aimed to directly compare the contemporary clinical outcomes of FG with those of non-perineal NSTIs using the NIS database.

## Methods

### Study design

We performed a retrospective cohort study using data from the NIS between 2016 and 2020. The NIS, which is part of the Healthcare Cost and Utilization Project (HCUP) sponsored by the Agency for Healthcare Research and Quality (AHRQ), is the largest all-payer inpatient care database in the United States and provides detailed data on inpatient hospital stays, including patient demographics, diagnoses, procedures, hospital characteristics, costs, and outcomes. NIS captures a stratified sample (20%) of all discharges from community and teaching hospitals, excluding rehabilitation and long-term acute care hospitals in the United States.

### Patient population

We included adult patients aged≥18 years who were hospitalized with a diagnosis of FG, gas gangrene, or necrotizing fasciitis (NSTI) and underwent at least one of the surgical procedures listed in the Supplemental Table. Pediatric patients (<18 yr) and those without documented debridement or amputation procedure codes were excluded.

Patients admitted for FG and other non-perineal NSTIs were identified using the following International Classification of Diseases, Tenth Revision, Clinical Modification (ICD-10-CM) codes: N49.3 (FG), N76.82 (Fournier disease of the vagina and vulva), M72.6 (necrotizing fasciitis), and A48.0 (gas gangrene), as described in previous studies.^5,10,11^ To accurately capture the intended patient population and distinguish between FG and non-perineal NSTIs, we stratified patients into two groups: (1) those who underwent perineal surgical debridement and (2) those with necrotizing fasciitis or gas gangrene who underwent non-perineal surgical interventions (Supplemental Table 2). This stratification ensured the inclusion of patients with NSTIs while maintaining a clear distinction between FG and non-perineal NSTIs.

### Variables and outcomes

We obtained patient and hospital characteristics such as age, sex, race/ethnicity, insurance status categorized by payer type, socioeconomic status represented by zip code income quartiles, hospital size, location, and teaching status. Comorbidities were assessed using All Patient Refined Diagnosis-Related Groups (APR-DRGs) and included 19 conditions such as alcohol abuse, drug abuse, and obesity (Supplemental Table 1). The outcomes of interest were in-hospital mortality, hospital LOS, cost, and home discharge.

### Statistical analysis

Categorical variables are reported as counts with percentages. Continuous variables are reported as median with interquartile range (IQR). P-values for the differences among the diagnosis groups were calculated using an ANOVA test for continuous variables and χ^2^ tests for categorical variables. We analyzed the difference in outcomes of interest between FG and non-perineal NSTIs, using a multivariable logistic regression for mortality and home discharge and a multivariable linear regression for LOS and total charges. Covariates were selected based on their significant association with the diagnosis groups. Adjusted odds ratio (aOR) with a 95% confidence interval (95% CI) was reported regarding the effect of diagnosis on mortality and home discharge. The adjusted mean difference with 95% CI was reported for the effect of diagnosis on LOS and total charges. A 2-sided *P* value<.05 was considered statistically significant. All analyses were conducted using SAS software (version 9.4, SAS Institute Inc., Cary, NC, USA). Institutional Review Board approval was not required as the NIS is a de-identified, publicly available data set that does not contain direct patient identifiers.

## Results

### Demographic and clinical characteristics

**Figure 1.**
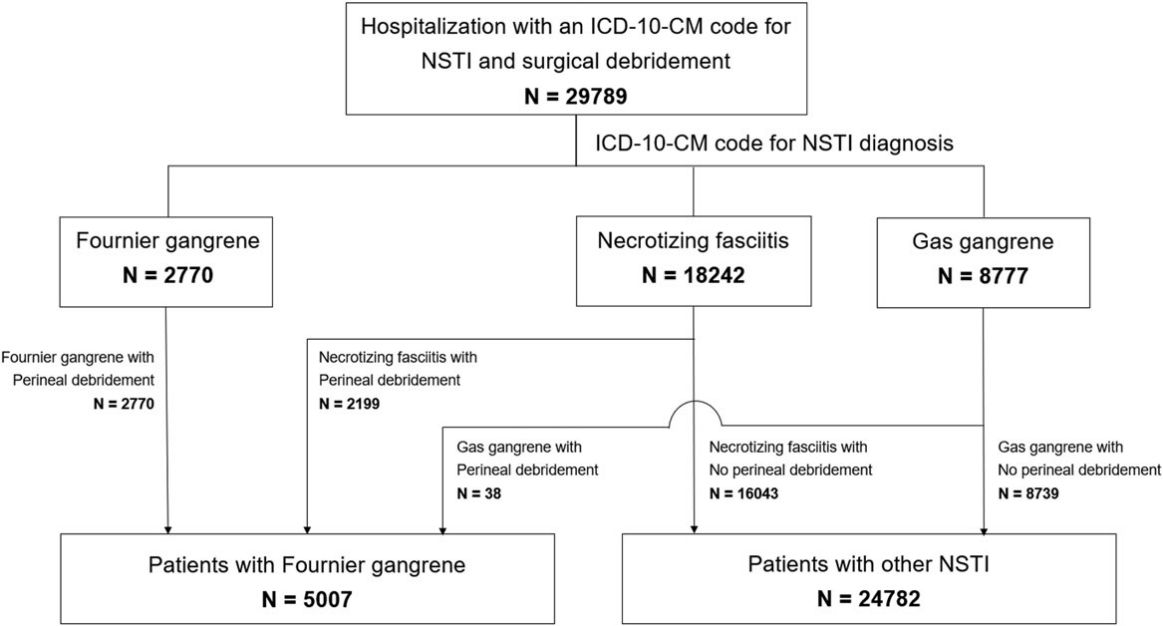
Consort diagram of the study cohort. Abbreviations: ICD-10-CM, International Classification of Diseases, Tenth Revision, Clinical Modification; NSTI, necrotizing soft tissue infection. *There was no patient with an ICD-10-CM code for Fournier disease of vagina and vulva who underwent surgical debridement.

From 2016 to 2020, the NIS database identified 29,789 admissions, of which 5,007 (16.8%) were for NSTI patients who underwent perineal surgical intervention and 24,782 (83.2%) for patients with necrotizing fasciitis or gas gangrene who underwent non-perineal surgical intervention (Figure 1). The patient and hospital characteristics and yearly trends are summarized in Table 1. Overall, the median age was 56, and the male gender was seen in 65.2%. While white race was dominant at 58.3%, followed by Black (21.4%). Medicare was the most common primary payer (38.8%), followed by Medicaid (25.0%). The distribution of patients based on the median household income quartile of their ZIP codes revealed a significant proportion residing in lower-income areas. Patients from the lowest income quartile (Quartile 1) comprised 38.9% (n = 11,264), followed by Quartile 2 with 26.9% (n = 7,792). Most patients received care at urban teaching hospitals, representing 75.8% (n = 22,577) of the total cohort. The highest proportion of patients were treated at hospitals located in the South, comprising 41.5% (n = 12,366). This was followed by hospitals in the Midwest (21.0%, n = 6,245), the West (20.8%, n = 6,197), and the Northeast (16.7%, n = 4,9819). Overall mortality (5.5% in 2016 and 5.6% in 2020) and median LOS (11 d in 2016 and 10 d in 2020) remained stable during the study period.

**Table 1.** Demographic and clinical characteristics by year

Characteristics		Overall	2016	2017	2018	2019	2020
Age, Years [IQR]		56 [47–65]	56 [46–64]	56 [45–64]	56 [47–65]	57 [47–65]	57 [47–65]
Sex	Male	19,425 (65.2%)	2,874 (62.4%)	3,184 (63.4%)	4,040 (66.1%)	4,364 (66.1%)	4,963 (66.7%)
	Female	10,357 (34.8%)	1,731 (37.6%)	1,835 (36.6%)	2,071 (33.9%)	2,243 (33.9%)	2,477 (33.3%)
Race	White	16,926 (58.3%)	2,700 (60.9%)	2,897 (59.2%)	3,435 (57.5%)	3,726 (57.7%)	4,168 (57.4%)
	Black	6,197 (21.4%)	929 (21.0%)	1,046 (21.4%)	1,253 (21.0%)	1,401 (21.7%)	1,568 (21.6%)
	Hispanic	4,146 (14.3%)	561 (12.7%)	660 (13.5%)	899 (15.0%)	940 (14.6%)	1,086 (14.9%)
	Asian or Pacific Islander	466 (1.6%)	66 (1.5%)	71 (1.5%)	115 (1.9%)	109 (1.7%)	105 (1.4%)
	Native American	514 (1.8%)	66 (1.5%)	90 (1.8%)	106 (1.8%)	108 (1.7%)	144 (2.0%)
	Other	769 (2.7%)	109 (2.5%)	127 (2.6%)	166 (2.8%)	172 (2.7%)	195 (2.7%)
Primary expected payer	Medicare	11,547 (38.8%)	1,707 (37.1%)	1,883 (37.6%)	2,426 (39.7%)	2,610 (39.5%)	2,921 (39.3%)
	Medicaid	7,439 (25.0%)	1,182 (25.7%)	1,305 (26.1%)	1,484 (24.3%)	1,567 (23.7%)	1,901 (25.6%)
	Private insurance	7,383 (24.8%)	1,192 (25.9%)	1,248 (24.9%)	1,513 (24.8%)	1,687 (25.6%)	1,743 (23.5%)

### Comparison between those with Fournier gangrene and those with non-perineal necrotizing soft tissue infections

Demographic and clinical characteristics stratified by FG and non-perineal NSTIs are summarized in Table 2. Both diagnostic groups exhibited similar distributions in age (56 [46–64] years in FG vs 57 [47–65] years in non-perineal NSTIs), sex (68.7% and 64.5% male), race (65.7% and 56.9% White), primary expected payer (37.3% and 39.1% Medicare), and median household income (38.1% and 39.0% in Quartile 1). The distribution of hospital characteristics was also similar between the groups, with most patients being hospitalized at large (57.8% and 52.8%) urban teaching hospitals (76.6% and 75.6%). Diabetes mellitus with chronic complications was common in both FG (55.0%) and non-perineal NSTIs (65.7%). Obesity was more prevalent in patients with FG (49.1%) compared to those with non-perineal NSTIs (31.2%) as a preexisting comorbidity. On the other hand, patients in the non-perineal NSTIs more frequently had substance use (3.2% in FG vs 9.0% in non-perineal NSTIs) and peripheral vascular disease (4.2% in FG vs 13.3% in non-perineal NSTIs). Unadjusted in-hospital mortality for admissions with a diagnosis of FG was 5.8%, compared to 5.4% for other non-perineal NSTIs.

**Table 2.** Demographic and clinical characteristics by diagnosis group

Characteristics		Overall	Fournier gangrene	Other NSTI
*Demographics*				
Age, Years [IQR]		56 [47–65]	56 [46–64]	57 [47–65]
Sex	Male	19,425 (65.2%)	3,436 (68.7%)	15,989 (64.5%)
	Female	10,357 (34.8%)	1,568 (31.3%)	8,789 (35.5%)
Race	White	16,926 (58.3%)	3,173 (65.7%)	13,753 (56.9%)
	Black	6,197 (21.4%)	840 (17.4%)	5,357 (22.1%)
	Hispanic	4,146 (14.3%)	567 (11.7%)	3,579 (14.8%)
	Asian or Pacific Islander	466 (1.6%)	66 (1.4%)	400 (1.7%)
	Native American	514 (1.8%)	75 (1.6%)	439 (1.8%)
	Other	769 (2.7%)	108 (2.2%)	661 (2.7%)
Primary expected payer	Medicare	11,547 (38.8%)	1,866 (37.3%)	9,681 (39.1%)
	Medicaid	7,439 (25.0%)	1,052 (21.0%)	6,387 (25.8%)
	Private insurance	7,383 (24.8%)	1,522 (30.4%)	5,861 (23.7%)
	Self-pay	2,283 (7.7%)	388 (7.8%)	1,895 (7.7%)
	No charge	205 (.7%)	29 (.6%)	176 (.7%)
	Other	886 (3.0%)	142 (2.8%)	744 (3.0%)
Median household income National Quartile for patient ZIP code	Quartile 1	11,264 (38.9%)	1,865 (38.1%)	9,399 (39.0%)
	Quartile 2	7,792 (26.9%)	1,367 (27.9%)	6,425 (26.7%)
	Quartile 3	6,145 (21.2%)	1,058 (21.6%)	5,087 (21.1%)
	Quartile 4	3,791 (13.1%)	608 (12.4%)	3,183 (13.2%)
Location/Teaching status of hospital	Rural	2,085 (7.0%)	334 (6.7%)	1,751 (7.1%)
	Urban nonteaching	5,127 (17.2%)	837 (16.7%)	4,290 (17.3%)
	Urban teaching	22,577 (75.8%)	3,836 (76.6%)	18,741 (75.6%)
Region of hospital	Northeast	4,981 (16.7%)	772 (15.4%)	4,209 (17.0%)
	Midwest	6,245 (21.0%)	1,168 (23.3%)	5,077 (20.5%)
	South	12,366 (41.5%)	2,196 (43.9%)	10,170 (41.0%)
	West	6,197 (20.8%)	871 (17.4%)	5,326 (21.5%)
Bed size of hospital	Small	5,070 (17.0%)	722 (14.4%)	4,348 (17.5%)
	Medium	8,744 (29.4%)	1,389 (27.7%)	7,355 (29.7%)
	Large	15,975 (53.6%)	2,896 (57.8%)	13,079 (52.8%)
*Clinical characteristics*				
Acquired immune deficiency syndrome		235 (.8%)	38 (.8%)	197 (.8%)
Alcohol abuse		1,450 (4.9%)	240 (4.8%)	1,210 (4.9%)
Leukemia		151 (.5%)	25 (.5%)	126 (.5%)
Lymphoma		140 (.5%)	26 (.5%)	114 (.5%)
Metastatic cancer		370 (1.2%)	120 (2.4%)	250 (1.0%)
Solid tumor without metastasis: in Situ		5 (.0%)	2 (.0%)	3 (.0%)
Solid tumor without metastasis: malignant		497 (1.7%)	119 (2.4%)	378 (1.5%)
Dementia		793 (2.7%)	103 (2.1%)	690 (2.8%)
Depression		3,336 (11.2%)	591 (11.8%)	2,745 (11.1%)
Diabetes with chronic complications		19,042 (63.9%)	2,753 (55.0%)	16,289 (65.7%)
Diabetes without chronic complications		2,112 (7.1%)	689 (13.8%)	1,423 (5.7%)
Drug abuse		2,377 (8.0%)	158 (3.2%)	2,219 (9.0%)
Hypertension: complicated		9,496 (31.9%)	1,296 (25.9%)	8,200 (33.1%)
Hypertension: uncomplicated		10,406 (34.9%)	1,965 (39.2%)	8,441 (34.1%)
Chronic pulmonary disease		4,518 (15.2%)	910 (18.2%)	3,608 (14.6%)
Obesity		10,183 (34.2%)	2,460 (49.1%)	7,723 (31.2%)
Peripheral vascular disease		3,517 (11.8%)	211 (4.2%)	3,306 (13.3%)
Hypothyroidism		2,137 (7.2%)	339 (6.8%)	1,798 (7.3%)
Other thyroid disorders		186 (.6%)	16 (.3%)	170 (.7%)
*Unadjusted outcomes*				
Mortality		1,634 (5.5%)	288 (5.8%)	1,346 (5.4%)
Length of stay, days [IQR]		10 [6–17]	11 [6–19]	10 [6–17]
Total charges [IQR]		114,295 [63,479–213,154]	128,110 [67,629–248,034]	112,190 [62,832–206,519]

Abbreviations: NSTI, necrotizing soft tissue infection; IQR, interquartile range.

### Adjusted analyses for in-hospital mortality, length of stay, cost, and home discharge

Selected covariates used in the multivariable regression model based on their significant association with the diagnoses included age, sex, race, primary expected payer, hospital region, metastatic cancer, solid tumor without metastasis: malignant, dementia, diabetes with chronic complications, diabetes without chronic complications, drug abuse, hypertension: complicated, hypertension: uncomplicated, chronic pulmonary disease, obesity, peripheral vascular disease, and other thyroid disorders. After adjusting for the baseline demographics and clinical and hospital characteristics, there was no significant difference in in-hospital mortality between FG and non-perineal NSTIs (aOR, 1.04; 95% CI, .90–1.20, Table 3). Hospitalization for perineal NSTI was associated with longer LOS by 1.99 days (95% CI, 1.53–2.46 d) and higher cost of hospital care by $37,809 (95% CI, $29,540–$46,077). The odds of home discharge were not significantly different between FG and non-perineal NSTIs (aOR, .97; 95% CI, .89–1.05).

**Table 3.** Multivariable logistic and linear regression analysis for mortality, home discharge, length of stay, and total charges between patients with fournier gangrene and other necrotizing soft tissue infections from 2016 to 2022^*^

Outcome comparison (FG compared to other NSTI)	Adjusted odds ratio (95% CI)	Adjusted mean difference (95% CI)	*P* value
Mortality	1.04 (.90, 1.20)	–	NS
Home discharge	.97 (.89, 1.05)	–	NS
Length of stay in days	**–**	**1.99 (1.53, 2.46)**	**<.001**
Total charges in U.S. dollars	**–**	**37,809 (29,540, 46 077)**	**<.001**

Abbreviations: FG, Fournier gangrene; NSTI, necrotizing soft tissue infection; CI, confidence interval; NS, Non-significant.

*Statistically significant numbers were highlighted in boldface type.

## Discussion

In this retrospective cohort study using the NIS database, we compared the clinical outcomes of perineal versus non-perineal NSTIs over five years (2016–2020). Approximately one in six patients had FG. In-hospital mortality rates remained similar for FG and other NSTIs during the study period, with an overall morality at 5.5%. Multivariable regression analysis demonstrated that, while in-hospital mortality was comparable between the two groups, FG was associated with a significantly longer LOS by approximately two days and higher hospital care costs by $38,399 compared to non-perineal NSTIs after adjusting for baseline demographic, clinical, and hospital characteristics.

The mortality risk did not differ between FG and other non-perineal NSTIs in this study. Over recent decades, the overall complexity of NSTI hospitalizations and the incidence of major complications have significantly increased. Between 1998 and 2010, the rate of significant complications rose from 30.9% to 48.2%, and the proportion of patients with multiple preexisting comorbidities (Elixhauser index>3) increased from 14.5% to 39.7%. However, during the same period, in-hospital mortality demonstrated a decreasing trend, declining from 9.0% to 4.9%.^10^ This declining trend in mortality associated with NSTIs has also been observed internationally.^12^ Our analysis found a comparable in-hospital mortality rate of 5.5% for NSTIs, with no significant downward trend over the five-year study period. These findings probably reflect improvements in clinical care, including early diagnosis of NSTIs, advancements in critical care, and enhanced wound care, which have enabled patients who previously would have died to survive NSTI hospitalization with complications.^10^ The favorable outcomes for FG reported at quaternary trauma and burn centers^7–9^ may not be generalizable nationwide due to potential selection bias in patient populations and the comprehensive, multidisciplinary care available at these facilities. Known predictors of mortality in NSTI include delays in surgery and illness severity (eg, APACHE scores), age, race, insurance status, and the number of major complications.^6,10,13–19^ These factors may have affected mortality more than the anatomical location of NSTIs (ie, FG vs non-perineal NSTIs). Additionally, certain monomicrobial presentations, such as necrotizing fasciitis with streptococcal toxic shock syndrome and clostridial gas gangrene, are more prevalent in extremity NSTIs and are associated with higher mortality rates.^20,21^ However, these subtypes constitute a minority of NSTIs and, therefore, likely have a limited impact on overall mortality trends.

On the other hand, we demonstrated that hospitalizations with a diagnosis of FG were associated with a longer LOS by two days and higher in-hospital healthcare costs compared to non-perineal NSTIs. The underlying factors driving this difference remain unclear. Previous studies have identified predictors of LOS in NSTIs, which include age, race, insurance type, Elixhauser comorbidity index, hyperbaric oxygen therapy, and the number of major complications.^10^ These variables, except for in-hospital complications, were adjusted for in the current analysis. Specifically for FG, the need for complex wound closure and urinary or fecal diversion have been recognized as the strongest predictors of prolonged LOS,^5^ which may have contributed to longer LOS and thus increased in-hospital healthcare costs in patients with FG. In contrast, some patients with limb NSTI may be ready for discharge to a rehabilitation facility relatively soon after undergoing amputation as the definitive source control surgery, potentially leading to a shorter hospital stay despite having similar illness severity on admission.

Historical trends in LOS for overall NSTIs have shown little change over time. Between 1998 and 2010, the median LOS decreased slightly from 13.3 to 11.9 days, though this difference was not statistically significant.^10^ In our study, the unadjusted median LOS in patients with FG (11 d [IQR: 6–19]) was longer compared to 9 days (IQR: 5–17) reported by Furr et al.^5^ This discrepancy may be attributable to differences in case selection. In our study, the FG cohort included patients with an ICD code for FG who underwent perineal debridement. In contrast, Furr et al included only patients with an ICD code for FG. Consequently, there is a possibility that a small proportion of patients in our FG cohort represented extremity or truncal NSTIs that secondarily extended to the perineum, requiring perineal debridement, thereby influencing LOS and associated healthcare costs. The higher healthcare costs observed in FG hospitalizations also remain unclear. Known determinants of NSTI-related healthcare costs include surgical procedures, ICU stay, and use of advanced therapies (eg, hyperbaric oxygen therapy or advanced wound care modalities).^10,11,22^ However, due to limitations of the NIS database, detailed data on resource utilization were unavailable. While the prolonged LOS in the FG group likely contributed to the increased costs, this alone may not fully explain the observed differences. Further research is warranted to better understand resource utilization and factors driving healthcare costs in FG management.

There was no significant difference in the odds of home discharge between patients with FG and those with non-perineal NSTIs. In a registry study conducted at a quaternary referral center, older age, female sex, non-white race, perineal involvement, and amputation were identified as predictors of discharge to a skilled care facility.^8^ While FG and non-perineal NSTIs frequently necessitate ongoing complex wound care after discharge, the specific needs for skilled nursing facilities may differ between these groups. Patients with FG often require specialized nursing care for managing suprapubic catheters or fecal diversion, whereas patients with NSTIs of the extremities may require rehabilitation services following amputation. These unique postdischarge needs highlight the importance of individualized care planning for patients recovering from NSTIs.

Our findings contradict the hypothesis that FG may have lower mortality due to a potentially less aggressive clinical course than other non-perineal NSTIs, as previously suggested by favorable outcomes reported in a limited number of smaller retrospective studies conducted at quaternary trauma and burn centers. Instead, our national database analysis demonstrates that the hospital course of FG is more complex and associated with more significant complications than non-perineal NSTIs. This discrepancy may potentially indicate nationwide gaps in the quality of care for FG outside of a limited number of specialized referral centers. It may highlight the significant practice variations in NSTI care previously reported.^6,23^ Further research is needed to elucidate what influences the improved prognosis of FG seen in those quaternary care centers and to disseminate and implement the necessary change.

Developing a dedicated institutional clinical pathway tailored specifically to FG, rather than grouping perineal and non-perineal NSTIs into “NSTI” guidance, may help optimize inpatient management, potentially reducing LOS and healthcare costs. To replicate favorable treatment outcomes of FG reported by specialized referral centers, early discharge and a shorter course of antibiotic therapy should be considered once surgical source control has been completed in patients with FG who had an uncomplicated hospital course without concurrent infectious complications necessitating prolonged antibiotic course (eg, osteomyelitis and undrained abscess). Multiple observational studies and their meta-analysis suggest the safety of a shorter duration of antibiotic treatment, which is now endorsed by the Surgical Infection Society guidelines.^7,9,24–27^ Long-term outcomes should be closely monitored following the implementation of a new institutional pathway to ensure promoting early discharge does not lead to an increase in readmissions, as unplanned readmissions after NSTI discharge are common and costly.^6,28^

This study has several limitations inherent to its retrospective nature based on a national database. First, while we utilized ICD-10 codes commonly used for NSTIs in prior studies, there is no validated data on the sensitivity and specificity of these codes for accurately identifying FG. However, our FG cohort specifically included cases with ICD-10 codes for debridement of the perineal area (Supplemental Table 2) during the index hospitalization, in addition to the primary ICD-10 code suggestive of FG. Second, illness severity indices such as APACHE II or SOFA scores,^29,30^ directly associated with in-hospital mortality, were unavailable. Third, the analyses did not include the timing of operative debridement (early vs delayed) and the number of operative debridement procedures. Fourth, wound care practices were not captured, including the type of wound care (eg, negative pressure wound therapy). Furthermore, there were no details on microbiology, the choice of antibiotic regimens or other medication relevant to FG including sodium-glucose cotransporter-2 inhibitors, which also could influence outcomes. The NIS also lacks data on readmissions and long-term follow-up after discharge, which limits our ability to evaluate long-term clinical outcomes and postdischarge care. Finally, the outcomes derived from the NIS reflect care patterns and healthcare logistics specific to acute care hospitals in the United States and may not be generalizable to other countries. Conducting similar studies in different countries would help determine whether the differences in LOS and healthcare costs observed in our study are attributable to U.S.-specific care practices. Despite these limitations, our study has several notable strengths. Utilizing a nationwide database encompassing diverse acute care hospitals, we identified national trends not apparent in small studies from a limited number of specialized quaternary care centers. Additionally, using multivariable analysis allowed us to adjust for potential confounders, providing a robust comparison between FG and non-perineal NSTIs.

In conclusion, this retrospective cohort study utilizing the NIS database revealed that FG is associated with a longer LOS and higher hospital care costs than non-perineal NSTIs. At the same time, in-hospital mortality and home discharge rates were comparable between the two groups. Currently, population-based studies comparing the clinical courses of these two distinct infections remain limited. A larger, well-designed, international multicenter cohort study is needed to determine whether FG has more favorable or unfavorable outcomes than non-perineal NSTIs in patients undergoing surgical interventions.

## Supporting information

10.1017/ash.2025.10084.sm001 Mitaka et al. supplementary material Mitaka et al. supplementary material

## Supplementary material

The supplementary material for this article can be found at https://doi.org/10.1017/ash.2025.10084
