# Supplementary material for: Fournier gangrene is associated with increased length of stay and higher healthcare costs compared to non-perineal necrotizing soft tissue infections: a retrospective analysis of the National Inpatient Sample (2016–2020)
Source: Antimicrob Steward Healthc Epidemiol. 2025 Aug 7;5(1):e178. doi: 10.1017/ash.2025.10084 (PMC12345050; doi:10.1017/ash.2025.10084)
Supplement: Mitaka et al. supplementary material [file S2732494X25100843sup001.docx]

**Supplemental document**

**Supplemental Table 1: Comorbidity data**

| **Characteristics** | **Overall** | **2016** | **2017** | **2018** | **2019** | **2020** |
| --- | --- | --- | --- | --- | --- | --- |
| Acquired Immune Deficiency Syndrome | 235 (0.8%) | 38 (0.8%) | 32 (0.6%) | 53 (0.9%) | 53 (0.8%) | 59 (0.8%) |
| Alcohol Abuse | 1450 (4.9%) | 234 (5.1%) | 275 (5.5%) | 293 (4.8%) | 302 (4.6%) | 346 (4.6%) |
| Leukemia | 151 (0.5%) | 21 (0.5%) | 20 (0.4%) | 36 (0.6%) | 31 (0.5%) | 43 (0.6%) |
| Lymphoma | 140 (0.5%) | 28 (0.6%) | 22 (0.4%) | 26 (0.4%) | 25 (0.4%) | 39 (0.5%) |
| Metastatic Cancer | 370 (1.2%) | 62 (1.3%) | 66 (1.3%) | 76 (1.2%) | 72 (1.1%) | 94 (1.3%) |
| Solid Tumor without Metastasis: in Situ | 5 (0.0%) | 0 (0.0%) | 1 (0.0%) | 2 (0.0%) | 1 (0.0%) | 1 (0.0%) |
| Solid Tumor without Metastasis: Malignant | 497 (1.7%) | 83 (1.8%) | 74 (1.5%) | 99 (1.6%) | 114 (1.7%) | 127 (1.7%) |
| Dementia | 793 (2.7%) | 128 (2.8%) | 104 (2.1%) | 158 (2.6%) | 179 (2.7%) | 224 (3.0%) |
| Depression | 3336 (11.2%) | 546 (11.8%) | 571 (11.4%) | 652 (10.7%) | 753 (11.4%) | 814 (10.9%) |
| Diabetes with Chronic Complications | 19042 (63.9%) | 2453 (53.2%) | 3005 (59.9%) | 3980 (65.1%) | 4424 (66.9%) | 5180 (69.6%) |
| Diabetes without Chronic Complications | 2112 (7.1%) | 580 (12.6%) | 388 (7.7%) | 388 (6.3%) | 360 (5.4%) | 396 (5.3%) |
| Drug abuse | 2377 (8.0%) | 368 (8.0%) | 430 (8.6%) | 496 (8.1%) | 497 (7.5%) | 586 (7.9%) |
| Hypertension: Complicated | 9496 (31.9%) | 1147 (24.9%) | 1525 (30.4%) | 2014 (33.0%) | 2220 (33.6%) | 2590 (34.8%) |
| Hypertension: Uncomplicated | 10406 (34.9%) | 1838 (39.9%) | 1799 (35.8%) | 2079 (34.0%) | 2244 (34.0%) | 2446 (32.9%) |
| Chronic Pulmonary Disease | 4518 (15.2%) | 756 (16.4%) | 754 (15.0%) | 877 (14.3%) | 1010 (15.3%) | 1121 (15.1%) |
| Obesity | 10183 (34.2%) | 1569 (34.0%) | 1772 (35.3%) | 1993 (32.6%) | 2226 (33.7%) | 2623 (35.3%) |
| Peripheral Vascular Disease | 3517 (11.8%) | 705 (15.3%) | 566 (11.3%) | 613 (10.0%) | 728 (11.0%) | 905 (12.2%) |
| Hypothyroidism | 2137 (7.2%) | 349 (7.6%) | 374 (7.5%) | 443 (7.2%) | 432 (6.5%) | 539 (7.2%) |
| Other Thyroid Disorders | 186 (0.6%) | 26 (0.6%) | 29 (0.6%) | 29 (0.5%) | 55 (0.8%) | 47 (0.6%) |

**Supplemental Table 2: International Classification of Diseases, Tenth Revision, Clinical Modification (ICD-10-CM) codes used in this study to capture perineal and non-perineal surgical interventions**

| Surgical Debridement | Destruction, Tendons | 0L5 | 0 Head and Neck Tendon  1 Shoulder Tendon, Right  2 Shoulder Tendon, Left  3 Upper Arm Tendon, Right  4 Upper Arm Tendon, Left  5 Lower Arm and Wrist  Tendon, Right  6 Lower Arm and Wrist  Tendon, Left  7 Hand Tendon, Right  8 Hand Tendon, Left  9 Trunk Tendon, Right  B Trunk Tendon, Left  C Thorax Tendon, Right  D Thorax Tendon, Left  F Abdomen Tendon, Right  G Abdomen Tendon, Left  H Perineum Tendon  J Hip Tendon, Right  K Hip Tendon, Left  L Upper Leg Tendon, Right  M Upper Leg Tendon, Left  N Lower Leg Tendon, Right  P Lower Leg Tendon, Left  Q Knee Tendon, Right  R Knee Tendon, Left  S Ankle Tendon, Right  T Ankle Tendon, Left  V Foot Tendon, Right  W Foot Tendon, Left |
| --- | --- | --- | --- |
|  | Division, Tendons | 0L8 | 0 Head and Neck Tendon  1 Shoulder Tendon, Right  2 Shoulder Tendon, Left  3 Upper Arm Tendon, Right  4 Upper Arm Tendon, Left  5 Lower Arm and Wrist  Tendon, Right  6 Lower Arm and Wrist  Tendon, Left  7 Hand Tendon, Right  8 Hand Tendon, Left  9 Trunk Tendon, Right  B Trunk Tendon, Left  C Thorax Tendon, Right  D Thorax Tendon, Left  F Abdomen Tendon, Right  G Abdomen Tendon, Left  H Perineum Tendon  J Hip Tendon, Right  K Hip Tendon, Left  L Upper Leg Tendon, Right  M Upper Leg Tendon, Left  N Lower Leg Tendon, Right  P Lower Leg Tendon, Left  Q Knee Tendon, Right  R Knee Tendon, Left  S Ankle Tendon, Right  T Ankle Tendon, Left  V Foot Tendon, Right  W Foot Tendon, Left |
|  | Drainage, Tendons | 0L9 | 0 Head and Neck Tendon  1 Shoulder Tendon, Right  2 Shoulder Tendon, Left  3 Upper Arm Tendon, Right  4 Upper Arm Tendon, Left  5 Lower Arm and Wrist  Tendon, Right  6 Lower Arm and Wrist  Tendon, Left  7 Hand Tendon, Right  8 Hand Tendon, Left  9 Trunk Tendon, Right  B Trunk Tendon, Left  C Thorax Tendon, Right  D Thorax Tendon, Left  F Abdomen Tendon, Right  G Abdomen Tendon, Left  H Perineum Tendon  J Hip Tendon, Right  K Hip Tendon, Left  L Upper Leg Tendon, Right  M Upper Leg Tendon, Left  N Lower Leg Tendon, Right  P Lower Leg Tendon, Left  Q Knee Tendon, Right  R Knee Tendon, Left  S Ankle Tendon, Right  T Ankle Tendon, Left  V Foot Tendon, Right  W Foot Tendon, Left |
|  | Excision, Tendons | 0LB | 0 Head and Neck Tendon  1 Shoulder Tendon, Right  2 Shoulder Tendon, Left  3 Upper Arm Tendon, Right  4 Upper Arm Tendon, Left  5 Lower Arm and Wrist  Tendon, Right  6 Lower Arm and Wrist  Tendon, Left  7 Hand Tendon, Right  8 Hand Tendon, Left  9 Trunk Tendon, Right  B Trunk Tendon, Left  C Thorax Tendon, Right  D Thorax Tendon, Left  F Abdomen Tendon, Right  G Abdomen Tendon, Left  H Perineum Tendon  J Hip Tendon, Right  K Hip Tendon, Left  L Upper Leg Tendon, Right  M Upper Leg Tendon, Left  N Lower Leg Tendon, Right  P Lower Leg Tendon, Left  Q Knee Tendon, Right  R Knee Tendon, Left  S Ankle Tendon, Right  T Ankle Tendon, Left  V Foot Tendon, Right  W Foot Tendon, Left |
|  | Extraction, Tendons | 0LD | 0 Head and Neck Tendon  1 Shoulder Tendon, Right  2 Shoulder Tendon, Left  3 Upper Arm Tendon, Right  4 Upper Arm Tendon, Left  5 Lower Arm and Wrist  Tendon, Right  6 Lower Arm and Wrist  Tendon, Left  7 Hand Tendon, Right  8 Hand Tendon, Left  9 Trunk Tendon, Right  B Trunk Tendon, Left  C Thorax Tendon, Right  D Thorax Tendon, Left  F Abdomen Tendon, Right  G Abdomen Tendon, Left  H Perineum Tendon  J Hip Tendon, Right  K Hip Tendon, Left  L Upper Leg Tendon, Right  M Upper Leg Tendon, Left  N Lower Leg Tendon, Right  P Lower Leg Tendon, Left  Q Knee Tendon, Right  R Knee Tendon, Left  S Ankle Tendon, Right  T Ankle Tendon, Left  V Foot Tendon, Right  W Foot Tendon, Left |
|  | Extirpation, Subcutaneous Tissue and Fascia | 0JC | 0 Subcutaneous Tissue and  Fascia, Scalp  1 Subcutaneous Tissue and  Fascia, Face  4 Subcutaneous Tissue and  Fascia, Right Neck  5 Subcutaneous Tissue and  Fascia, Left Neck  6 Subcutaneous Tissue and  Fascia, Chest  7 Subcutaneous Tissue and  Fascia, Back  8 Subcutaneous Tissue and  Fascia, Abdomen  9 Subcutaneous Tissue and  Fascia, Buttock  B Subcutaneous Tissue and  Fascia, Perineum  C Subcutaneous Tissue and  Fascia, Pelvic Region  D Subcutaneous Tissue and  Fascia, Right Upper Arm  F Subcutaneous Tissue and  Fascia, Left Upper Arm  G Subcutaneous Tissue and  Fascia, Right Lower Arm  H Subcutaneous Tissue and  Fascia, Left Lower Arm  J Subcutaneous Tissue and  Fascia, Right Hand  K Subcutaneous Tissue and  Fascia, Left Hand  L Subcutaneous Tissue and  Fascia, Right Upper Leg  M Subcutaneous Tissue and  Fascia, Left Upper Leg  N Subcutaneous Tissue and  Fascia, Right Lower Leg  P Subcutaneous Tissue and  Fascia, Left Lower Leg  Q Subcutaneous Tissue and  Fascia, Right Foot  R Subcutaneous Tissue and  Fascia, Left Foot |
|  | Drainage, Skin and Breast | 0H9 | 0 Skin, Scalp  1 Skin, Face  4 Skin, Neck  5 Skin, Chest  6 Skin, Back  7 Skin, Abdomen  8 Skin, Buttock  9 Skin, Perineum  A Skin, Inguinal  B Skin, Right Upper Arm  C Skin, Left Upper Arm  D Skin, Right Lower Arm  E Skin, Left Lower Arm  F Skin, Right Hand  G Skin, Left Hand  H Skin, Right Upper Leg  J Skin, Left Upper Leg  K Skin, Right Lower Leg  L Skin, Left Lower Leg  M Skin, Right Foot  N Skin, Left Foot |
|  | Excision, Skin and Breast | 0HB | 0 Skin, Scalp  1 Skin, Face  4 Skin, Neck  5 Skin, Chest  6 Skin, Back  7 Skin, Abdomen  8 Skin, Buttock  9 Skin, Perineum  A Skin, Inguinal  B Skin, Right Upper Arm  C Skin, Left Upper Arm  D Skin, Right Lower Arm  E Skin, Left Lower Arm  F Skin, Right Hand  G Skin, Left Hand  H Skin, Right Upper Leg  J Skin, Left Upper Leg  K Skin, Right Lower Leg  L Skin, Left Lower Leg  M Skin, Right Foot  N Skin, Left Foot |
|  | Destruction, Skin and Breast | 0H5 | 0 Skin, Scalp  1 Skin, Face  4 Skin, Neck  5 Skin, Chest  6 Skin, Back  7 Skin, Abdomen  8 Skin, Buttock  9 Skin, Perineum  A Skin, Inguinal  B Skin, Right Upper Arm  C Skin, Left Upper Arm  D Skin, Right Lower Arm  E Skin, Left Lower Arm  F Skin, Right Hand  G Skin, Left Hand  H Skin, Right Upper Leg  J Skin, Left Upper Leg  K Skin, Right Lower Leg  L Skin, Left Lower Leg  M Skin, Right Foot  N Skin, Left Foot |
|  | Division, Skin and Breast | 0H8 | 0 Skin, Scalp  1 Skin, Face  4 Skin, Neck  5 Skin, Chest  6 Skin, Back  7 Skin, Abdomen  8 Skin, Buttock  9 Skin, Perineum  A Skin, Inguinal  B Skin, Right Upper Arm  C Skin, Left Upper Arm  D Skin, Right Lower Arm  E Skin, Left Lower Arm  F Skin, Right Hand  G Skin, Left Hand  H Skin, Right Upper Leg  J Skin, Left Upper Leg  K Skin, Right Lower Leg  L Skin, Left Lower Leg  M Skin, Right Foot  N Skin, Left Foot |
|  | Destruction, Subcutaneous Tissue and Fascia | 0J5 | 0 Subcutaneous Tissue and  Fascia, Scalp  1 Subcutaneous Tissue and  Fascia, Face  4 Subcutaneous Tissue and  Fascia, Right Neck  5 Subcutaneous Tissue and  Fascia, Left Neck  6 Subcutaneous Tissue and  Fascia, Chest  7 Subcutaneous Tissue and  Fascia, Back  8 Subcutaneous Tissue and  Fascia, Abdomen  9 Subcutaneous Tissue and  Fascia, Buttock  B Subcutaneous Tissue and  Fascia, Perineum  C Subcutaneous Tissue and  Fascia, Pelvic Region  D Subcutaneous Tissue and  Fascia, Right Upper Arm  F Subcutaneous Tissue and  Fascia, Left Upper Arm  G Subcutaneous Tissue and  Fascia, Right Lower Arm  H Subcutaneous Tissue and  Fascia, Left Lower Arm  J Subcutaneous Tissue and  Fascia, Right Hand  K Subcutaneous Tissue and  Fascia, Left Hand  L Subcutaneous Tissue and  Fascia, Right Upper Leg  M Subcutaneous Tissue and  Fascia, Left Upper Leg  N Subcutaneous Tissue and  Fascia, Right Lower Leg  P Subcutaneous Tissue and  Fascia, Left Lower Leg  Q Subcutaneous Tissue and  Fascia, Right Foot  R Subcutaneous Tissue and  Fascia, Left Foot |
|  | Division, Subcutaneous Tissue and Fascia | 0J8 | 0 Subcutaneous Tissue and  Fascia, Scalp  1 Subcutaneous Tissue and  Fascia, Face  4 Subcutaneous Tissue and  Fascia, Right Neck  5 Subcutaneous Tissue and  Fascia, Left Neck  6 Subcutaneous Tissue and  Fascia, Chest  7 Subcutaneous Tissue and  Fascia, Back  8 Subcutaneous Tissue and  Fascia, Abdomen  9 Subcutaneous Tissue and  Fascia, Buttock  B Subcutaneous Tissue and  Fascia, Perineum  C Subcutaneous Tissue and  Fascia, Pelvic Region  D Subcutaneous Tissue and  Fascia, Right Upper Arm  F Subcutaneous Tissue and  Fascia, Left Upper Arm  G Subcutaneous Tissue and  Fascia, Right Lower Arm  H Subcutaneous Tissue and  Fascia, Left Lower Arm  J Subcutaneous Tissue and  Fascia, Right Hand  K Subcutaneous Tissue and  Fascia, Left Hand  L Subcutaneous Tissue and  Fascia, Right Upper Leg  M Subcutaneous Tissue and  Fascia, Left Upper Leg  N Subcutaneous Tissue and  Fascia, Right Lower Leg  P Subcutaneous Tissue and  Fascia, Left Lower Leg  Q Subcutaneous Tissue and  Fascia, Right Foot  R Subcutaneous Tissue and  Fascia, Left Foot  S Subcutaneous Tissue and  Fascia, Head and Neck  T Subcutaneous Tissue and  Fascia, Trunk  V Subcutaneous Tissue and  Fascia, Upper Extremity  W Subcutaneous Tissue and  Fascia, Lower Extremity |
|  | Drainage, Subcutaneous Tissue and Fascia | 0J9 | 0 Subcutaneous Tissue and  Fascia, Scalp  1 Subcutaneous Tissue and  Fascia, Face  4 Subcutaneous Tissue and  Fascia, Right Neck  5 Subcutaneous Tissue and  Fascia, Left Neck  6 Subcutaneous Tissue and  Fascia, Chest  7 Subcutaneous Tissue and  Fascia, Back  8 Subcutaneous Tissue and  Fascia, Abdomen  9 Subcutaneous Tissue and  Fascia, Buttock  B Subcutaneous Tissue and  Fascia, Perineum  C Subcutaneous Tissue and  Fascia, Pelvic Region  D Subcutaneous Tissue and  Fascia, Right Upper Arm  F Subcutaneous Tissue and  Fascia, Left Upper Arm  G Subcutaneous Tissue and  Fascia, Right Lower Arm  H Subcutaneous Tissue and  Fascia, Left Lower Arm  J Subcutaneous Tissue and  Fascia, Right Hand  K Subcutaneous Tissue and  Fascia, Left Hand  L Subcutaneous Tissue and  Fascia, Right Upper Leg  M Subcutaneous Tissue and  Fascia, Left Upper Leg  N Subcutaneous Tissue and  Fascia, Right Lower Leg  P Subcutaneous Tissue and  Fascia, Left Lower Leg  Q Subcutaneous Tissue and  Fascia, Right Foot  R Subcutaneous Tissue and  Fascia, Left Foot |
|  | Excision, Subcutaneous Tissue and Fascia | 0JB | 0 Subcutaneous Tissue and  Fascia, Scalp  1 Subcutaneous Tissue and  Fascia, Face  4 Subcutaneous Tissue and  Fascia, Right Neck  5 Subcutaneous Tissue and  Fascia, Left Neck  6 Subcutaneous Tissue and  Fascia, Chest  7 Subcutaneous Tissue and  Fascia, Back  8 Subcutaneous Tissue and  Fascia, Abdomen  9 Subcutaneous Tissue and  Fascia, Buttock  B Subcutaneous Tissue and  Fascia, Perineum  C Subcutaneous Tissue and  Fascia, Pelvic Region  D Subcutaneous Tissue and  Fascia, Right Upper Arm  F Subcutaneous Tissue and  Fascia, Left Upper Arm  G Subcutaneous Tissue and  Fascia, Right Lower Arm  H Subcutaneous Tissue and  Fascia, Left Lower Arm  J Subcutaneous Tissue and  Fascia, Right Hand  K Subcutaneous Tissue and  Fascia, Left Hand  L Subcutaneous Tissue and  Fascia, Right Upper Leg  M Subcutaneous Tissue and  Fascia, Left Upper Leg  N Subcutaneous Tissue and  Fascia, Right Lower Leg  P Subcutaneous Tissue and  Fascia, Left Lower Leg  Q Subcutaneous Tissue and  Fascia, Right Foot  R Subcutaneous Tissue and  Fascia, Left Foot |
|  | Removal, Subcutaneous Tissue and Fascia | 0JP | S Subcutaneous Tissue and  Fascia, Head and Neck  T Subcutaneous Tissue and  Fascia, Trunk  V Subcutaneous Tissue and  Fascia, Upper Extremity  W Subcutaneous Tissue and  Fascia, Lower Extremity |
|  | Inspection, Subcutaneous Tissue and Fascia | 0JJ | S Subcutaneous Tissue and  Fascia, Head and Neck  T Subcutaneous Tissue and  Fascia, Trunk  V Subcutaneous Tissue and  Fascia, Upper Extremity  W Subcutaneous Tissue and  Fascia, Lower Extremity |
|  | Release, Subcutaneous Tissue and Fascia | 0JN | 0 Subcutaneous Tissue and  Fascia, Scalp  1 Subcutaneous Tissue and  Fascia, Face  4 Subcutaneous Tissue and  Fascia, Right Neck  5 Subcutaneous Tissue and  Fascia, Left Neck  6 Subcutaneous Tissue and  Fascia, Chest  7 Subcutaneous Tissue and  Fascia, Back  8 Subcutaneous Tissue and  Fascia, Abdomen  9 Subcutaneous Tissue and  Fascia, Buttock  B Subcutaneous Tissue and  Fascia, Perineum  C Subcutaneous Tissue and  Fascia, Pelvic Region  D Subcutaneous Tissue and  Fascia, Right Upper Arm  F Subcutaneous Tissue and  Fascia, Left Upper Arm  G Subcutaneous Tissue and  Fascia, Right Lower Arm  H Subcutaneous Tissue and  Fascia, Left Lower Arm  J Subcutaneous Tissue and  Fascia, Right Hand  K Subcutaneous Tissue and  Fascia, Left Hand  L Subcutaneous Tissue and  Fascia, Right Upper Leg  M Subcutaneous Tissue and  Fascia, Left Upper Leg  N Subcutaneous Tissue and  Fascia, Right Lower Leg  P Subcutaneous Tissue and  Fascia, Left Lower Leg  Q Subcutaneous Tissue and  Fascia, Right Foot  R Subcutaneous Tissue and  Fascia, Left Foot |
|  | Revision, Subcutaneous Tissue and Fascia | 0JW | S Subcutaneous Tissue and  Fascia, Head and Neck  T Subcutaneous Tissue and  Fascia, Trunk  V Subcutaneous Tissue and  Fascia, Upper Extremity  W Subcutaneous Tissue and  Fascia, Lower Extremity |
|  | Destruction, Muscles | 0K5 | 0 Head Muscle  1 Facial Muscle  2 Neck Muscle, Right  3 Neck Muscle, Left  5 Shoulder Muscle, Right  6 Shoulder Muscle, Left  7 Upper Arm Muscle, Right  8 Upper Arm Muscle, Left  9 Lower Arm and Wrist  Muscle, Right  B Lower Arm and Wrist  Muscle, Left  C Hand Muscle, Right  D Hand Muscle, Left  F Trunk Muscle, Right  G Trunk Muscle, Left  H Thorax Muscle, Right  J Thorax Muscle, Left  K Abdomen Muscle, Right  L Abdomen Muscle, Left  M Perineum Muscle  N Hip Muscle, Right  P Hip Muscle, Left  Q Upper Leg Muscle, Right  R Upper Leg Muscle, Left  S Lower Leg Muscle, Right  T Lower Leg Muscle, Left  V Foot Muscle, Right  W Foot Muscle, Left |
|  | Division, Muscles | 0K8 | 0 Head Muscle  1 Facial Muscle  2 Neck Muscle, Right  3 Neck Muscle, Left  5 Shoulder Muscle, Right  6 Shoulder Muscle, Left  7 Upper Arm Muscle, Right  8 Upper Arm Muscle, Left  9 Lower Arm and Wrist  Muscle, Right  B Lower Arm and Wrist  Muscle, Left  C Hand Muscle, Right  D Hand Muscle, Left  F Trunk Muscle, Right  G Trunk Muscle, Left  H Thorax Muscle, Right  J Thorax Muscle, Left  K Abdomen Muscle, Right  L Abdomen Muscle, Left  M Perineum Muscle  N Hip Muscle, Right  P Hip Muscle, Left  Q Upper Leg Muscle, Right  R Upper Leg Muscle, Left  S Lower Leg Muscle, Right  T Lower Leg Muscle, Left  V Foot Muscle, Right  W Foot Muscle, Left |
|  | Excision, Muscles | 0KB | 0 Head Muscle  1 Facial Muscle  2 Neck Muscle, Right  3 Neck Muscle, Left  5 Shoulder Muscle, Right  6 Shoulder Muscle, Left  7 Upper Arm Muscle, Right  8 Upper Arm Muscle, Left  9 Lower Arm and Wrist  Muscle, Right  B Lower Arm and Wrist  Muscle, Left  C Hand Muscle, Right  D Hand Muscle, Left  F Trunk Muscle, Right  G Trunk Muscle, Left  H Thorax Muscle, Right  J Thorax Muscle, Left  K Abdomen Muscle, Right  L Abdomen Muscle, Left  M Perineum Muscle  N Hip Muscle, Right  P Hip Muscle, Left  Q Upper Leg Muscle, Right  R Upper Leg Muscle, Left  S Lower Leg Muscle, Right  T Lower Leg Muscle, Left  V Foot Muscle, Right  W Foot Muscle, Left |
|  | Resection, Muscles | 0KT | 0 Head Muscle  1 Facial Muscle  2 Neck Muscle, Right  3 Neck Muscle, Left  5 Shoulder Muscle, Right  6 Shoulder Muscle, Left  7 Upper Arm Muscle, Right  8 Upper Arm Muscle, Left  9 Lower Arm and Wrist  Muscle, Right  B Lower Arm and Wrist  Muscle, Left  C Hand Muscle, Right  D Hand Muscle, Left  F Trunk Muscle, Right  G Trunk Muscle, Left  H Thorax Muscle, Right  J Thorax Muscle, Left  K Abdomen Muscle, Right  L Abdomen Muscle, Left  M Perineum Muscle  N Hip Muscle, Right  P Hip Muscle, Left  Q Upper Leg Muscle, Right  R Upper Leg Muscle, Left  S Lower Leg Muscle, Right  T Lower Leg Muscle, Left  V Foot Muscle, Right  W Foot Muscle, Left |
| Amputation | Detachment, Upper Extremities | 0X6 | 0 Forequarter, Right  1 Forequarter, Left  2 Shoulder Region, Right  3 Shoulder Region, Left  B Elbow Region, Right  C Elbow Region, Left  8 Upper Arm, Right  9 Upper Arm, Left  D Lower Arm, Right  F Lower Arm, Left  J Hand, Right  K Hand, Left  L Thumb, Right  M Thumb, Left  N Index Finger, Right  P Index Finger, Left  Q Middle Finger, Right  R Middle Finger, Left  S Ring Finger, Right  T Ring Finger, Left  V Little Finger, Right  W Little Finger, Left |
|  | Detachment, Lower Extremities | 0Y6 | 2 Hindquarter, Right  3 Hindquarter, Left  4 Hindquarter, Bilateral  7 Femoral Region, Right  8 Femoral Region, Left  F Knee Region, Right  G Knee Region, Left  C Upper Leg, Right  D Upper Leg, Left  H Lower Leg, Right  J Lower Leg, Left  M Foot, Right  N Foot, Left  P 1st Toe, Right  Q 1st Toe, Left  R 2nd Toe, Right  S 2nd Toe, Left  T 3rd Toe, Right  U 3rd Toe, Left  V 4th Toe, Right  W 4th Toe, Left  X 5th Toe, Right  Y 5th Toe, Left |
